# Children and young people’s experiences of receiving group metacognitive therapy: Thematic analysis of a transdiagnostic treatment for common mental health problems

**DOI:** 10.3389/fpsyt.2025.1671086

**Published:** 2025-10-09

**Authors:** Adrian Wells, Noor Nasseri, Karin Carter, Lora Capobianco

**Affiliations:** ^1^ Division of Psychology and Mental Health, School of Psychological Sciences, Faculty of Biology, Medicine and Health, The University of Manchester, Manchester, United Kingdom; ^2^ Research and Innovation, Greater Manchester Mental Health NHS Foundation Trust, Manchester, United Kingdom

**Keywords:** metacognitive therapy, children and adolescents, common mental health problems, transdiagnostic treatment, anxiety, depression, group therapy

## Abstract

**Background:**

Mental health problems in children and young people (CYP) are increasing with a pressing need for more effective treatments. However, the development of psychological interventions seldom explores young patients experiences of treatment, which is crucial in understanding factors influencing the uptake, impact and validity of therapy. We aimed to explore for the first time CYP experiences of how they received group metacognitive therapy for anxiety disorders and depression.

**Methods:**

A qualitative study was embedded in a larger feasibility RCT (n=95) comparing group-based MCT with treatment as usual. Seventeen CYP aged 11-17yrs who had been part of the group-MCT arm consented to participate. Interviews were semi-structured, open-ended and followed an a-priori guide. Coding and analysis adhered to guidelines for reflexive thematic analysis.

**Results:**

Three overarching themes emerged in patient experiences: treatment fidelity, treatment delivery, and experiences of homework. Treatment fidelity had two subthemes: i) treatment receipt- which included patients understanding of MCT and performance of MCT techniques during the intervention, and ii) treatment enactment- which included performance of MCT techniques in applied settings, plus perceived benefits of treatment. Treatment delivery included two subthemes; i) format of therapy and, ii) therapist characteristics.

**Conclusions:**

The results support the use of MCT in children and young people with mixed anxiety disorders and depression. Patients reported understanding the treatment rationale and benefiting from the intervention. They described, consistent with purported mechanisms, how treatment helped them make a shift in beliefs about thoughts and see worry as powerless and under personal control. Patients described an ability to apply specific techniques in real-life settings, despite noting major challenges with homework compliance. The results emphasised areas that might be improved and important recommendations are made for MCT delivery and practise in children and adolescents.

## Introduction

Mental health problems among children and young people (CYP) have become a significant and global health concern. Common mental health issues, such as anxiety and depression, affect one in five CYP in the UK (1) and profoundly impact the developmental trajectory and wellbeing of young individuals (2, 3). The number of referrals for mental health problems has increased rapidly by 11.7%, with 120,000 referrals made per month in 2024 in the UK (4). As such, there has been a significant increase in the demand for mental health provision and for effective interventions to treat mental health problems in this population.

Metacognitive therapy (MCT) (5, 6) is an empirically supported psychological therapy for mental health problems based on the self-regulatory executive function model of psychological disorder (7, 8). Evidence of the effectiveness of MCT is growing, with recent meta-analyses suggesting it can outperform traditional cognitive-behavioural therapy (CBT) approaches in adult mental health (9, 10). There is a burgeoning interest in applying MCT to children and adolescents (11) with preliminary studies indicating promising outcomes (12–14).

Unlike content-focused disorder-specific CBT approaches, MCT targets an underlying common (i.e. transdiagnostic) processing style termed the cognitive attentional syndrome (CAS) which is considered to maintain most psychological disorders. The CAS is characterised by worry, rumination, sustained attention on threat, and maladaptive coping strategies that interfere with emotional regulation. The CAS is related to biases in metacognition, amongst which beliefs about the uncontrollability of perseverative thinking (e.g. worry/rumination) are universal factors (6, 7). As MCT is a transdiagnostic intervention, a wide range of mood, anxiety and stress symptoms can be treated with the same methods and at the same time, offering an advantage over other approaches which tend to focus on disorder-specific protocols and require the most pressing disorder to be treated first. While previous MCT research in CYP has focused on evaluating the metacognitive model (15, 16) and on preliminary treatment outcomes (12–14), studies have yet to examine young people’s phenomenological experiences of undergoing metacognitive therapy. Such experiences are important in understanding how well MCT meets patient needs, whether treatment mechanisms are correctly comprehended and for discovering barriers and facilitators to uptake within a population that can be challenging to engage.

Systematic reviews have evaluated patients’ experiences of several other psychological therapies (17, 18). In a qualitative meta-synthesis, including 11 studies of young people’s experiences of psychoanalytic therapy, four themes were identified relating to: learning to navigate roles during therapy, the importance of the therapeutic relationship, experiencing psychoanalysis as emotionally ‘painful’, and the perceived impact of therapy being difficult to gauge (17). In a systematic review and meta-synthesis of youth experiences of trauma-focused CBT, children and adolescents were often unclear about what to expect from treatment and concerned about incompatibility with their therapist. However, reports indicated how this might be addressed through early consideration and efforts to strengthen the therapeutic alliance. Once underway, treatment was viewed as a place of refuge and validation, aided by therapist competence and confidentiality (18). The literature provides valuable insights into the wide-ranging nature of subjective experiences, some of which are common across therapeutic approaches such as the importance of the therapeutic alliance, and young people’s expectations of the treatment and the role they will play during therapy, whilst others are more specific. In each case emergent themes point to important issues that could act as barriers to reliable use, impact and uptake of treatment.

Exploring patient attitudes and experiences of mental health interventions is crucial in helping researchers and practitioners to understand factors that influence the uptake and impact of treatment. This is particularly important in the context of interventions involving CYP, where patient opinions and perspectives are under-represented because of reliance on parent-report or teacher-report methods and measurements that have been developed by adult researchers with little or no CYP involvement or feedback (17, 19). Since treatment outcomes are often quantified using symptom measures and are based on assessing the perceptions of parents and observers (17, 19), it is important to understand the ways in which young people themselves experience benefit. The young person’s voice in treatment development and evaluation is rarely sought, despite the important contribution it can bring to understanding the ‘goodness of fit’ of a treatment approach and acceptability of therapy and ways it might be improved. Young people can reliably report on their experiences of mental health, and their views can diverge in important ways from adults (20, 21).

The present study aimed to explore for the first time youth experiences of participating in group metacognitive therapy for anxiety disorders and depression. The study was part of an NIHR-funded randomised feasibility trial of metacognitive therapy versus treatment as usual conducted within child and adolescent mental health services in the National Health Service. We aimed to explore children and adolescents understanding, engagement with and effects associated with MCT in preparation for subsequent trials.

## Methods

### Design

Semi-structured interviews were conducted with young people who received group-metacognitive therapy (MCT) as part of the Youth Metacognitive Therapy (YoMeta) trial (11). Ethical approval for the study was granted by the North West Greater Manchester East Research Ethics Committee (REC ref: 21/NW/032).

### Participants

Children and young people who received at least one MCT session, including dropouts were invited to take part in the interviews, to ensure we captured a range of views and any barriers to engaging in a group intervention. For details of the inclusion and exclusion criteria, please refer to the published study protocol (11).

A total of 95 patients were recruited to the main RCT and allocation produced good balance between arms with numbers in each of n=48 and n=47. All patients allocated to receive group-MCT were invited to take part in a qualitative interview. Seventeen such patients agreed to participate and provided written or verbal consent. CYP under the age of 16 provided assent and parental consent was obtained. The majority of the sample (n = 15, 88%) were female, with an average age of 14.12 years (range 11-17; *SD* = 1.50). The average number of MCT sessions attended was 6.76 (out of 8 sessions), with fifteen (88%) of the patients receiving a minimum likely effective dose noted in previous studies as 4 or more sessions (22). See **Table 1** for participant characteristics. Note that one participant had reached the age of 17 at the time of interview, but the age range during the RCT was 11-16.

**Table 1:** Participant characteristics.

Participant Number	Site Location	Reason for seeking treatment	Sex	Age at interview	Number of MCT sessions attended
1	A	GAD	Female	13	8
2	B	GAD, SAD & Depression	Female	16	8
3	A	Depression	Female	13	8
4	B	Panic & Depression	Female	17	3
5	A	GAD & Depression	Female	13	1
6	B	GAD, SAD, OCD	Female	13	8
7	A	Anxiety NOS	Female	15	6
8	A	OCD	Male	11	6
9	A	Anxiety NOS	Female	16	8
10	B	Anxiety NOS	Female	15	8
11	B	Anxiety NOS	Female	13	8
12	C	OCD	Female	14	8
13	A	Anxiety NOS	Female	14	8
14	A	SAD	Female	15	7
15	C	Anxiety NOS	Male	13	8
16	C	Anxiety NOS & Depression	Female	14	4
17	C	PTSD	Female	15	8

A, Central Manchester; B, South Manchester; C, North Manchester; GAD, Generalized Anxiety Disorder; SAD, Social Anxiety Disorder; OCD, Obsessive Compulsive Disorder; NOS, Not Otherwise Specified; PTSD, Post- Traumatic Stress Disorder.

### Data collection

All interviews were conducted individually and were structured using an interview guide. The interviews took place either online or in the patient’s own home and were conducted by one of three female qualitative researchers between November 2022 to October 2023. Patients were asked about aspects of the intervention they found challenging to understand or to engage in, aspects of the intervention that they enjoyed, and their experiences of complying with the intervention and with homework. The interviews lasted an average of 27.43 minutes, ranging from 7.07 minutes to 47.22 minutes, producing a total of 466.33 minutes of data. Interviews were audio-recorded and transcribed verbatim. The transcripts were uploaded and managed electronically using NVIVO 14 qualitative data management software.

### Data analysis

Data were analysed using reflexive thematic analysis (TA), as described by Braun and Clarke (23, 24). Reflexive TA involves six phases of qualitative analysis that aim to identify and interpret patterns of shared meaning across a dataset (25). These six phases include (1) data familiarisation, (2) coding, (3) generating initial themes, (4) developing and reviewing themes, (5) naming, refining, and defining themes, and (6) creating the report.

The analysis began with one author (NN) familiarising herself with the dataset by reading and re-reading the transcripts multiple times and annotating any initial points of interest. Codes were then developed throughout the data using an inductive-semantic approach to minimize the influence of pre-existing theory and research. Coding was an interactive process whereby NN identified and labelled important features of the data relevant to the aims of the study. Initial themes were then generated by combining codes which shared similar meanings. Following this, AW led a series of discussions with NN, LC, and KC whereby codes and themes were further developed, reviewed, and refined, and were examined for potential discrepancies. A thematic map was designed to assess the representativeness of the thematic structure of CYPs MCT experiences. This visual representation revealed shared meanings across different themes and subthemes. As a result, multiple subthemes were aggregated together and following three versions of the thematic structure, one final thematic map with three overarching themes was agreed. The labelling of themes was informed by the research team’s experience, with some of the narratives reflecting issues pertinent to treatment fidelity/adherence, prompting the team to refer to a fidelity framework (26) to inform aggregation and labelling of the thematic structure. The fidelity framework (26) used encompasses five domains; study design, provider training, treatment delivery, treatment receipt, and treatment enactment. The current analysis drew on two domains: treatment receipt and treatment enactment that appeared present in patient narrative content. Treatment receipt is a domain that encompasses whether the participant understood the treatment, ability to demonstrate knowledge of skills learned in therapy as well as the ability to use such skills. Treatment enactment on the other hand focuses on whether or not the skills learned are implemented appropriately and have the desired effect on the relevant outcomes.

## Results

**Figure 1:**
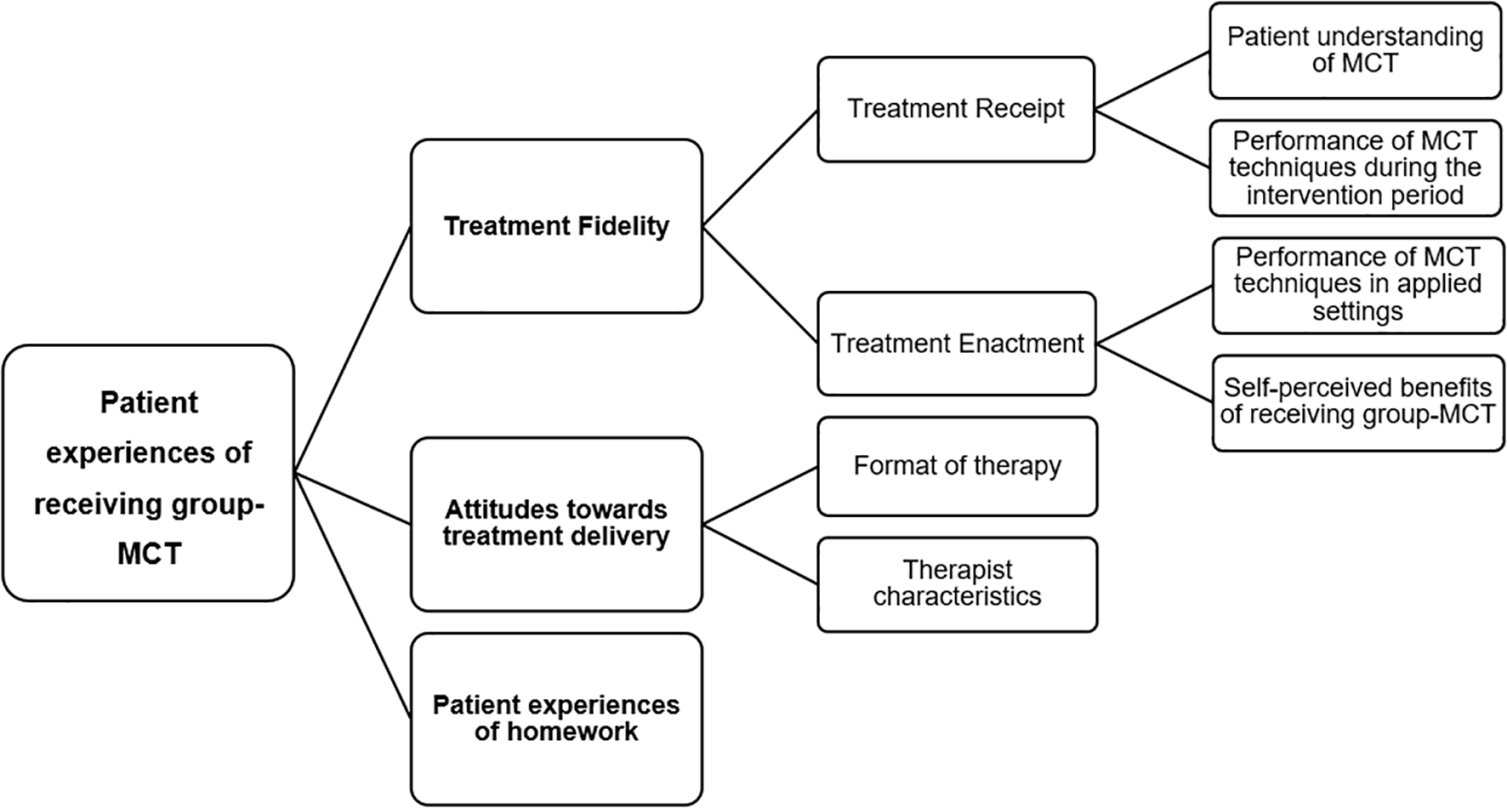
Patients’ views and experiences of receiving group-MCT.

Three overarching themes were identified and labelled as: (1) Treatment fidelity, (2) Attitudes towards treatment delivery, and (3) Patient experiences of homework (see **Figure 1**).

### Theme 1: treatment fidelity

Treatment fidelity concerned patients’ experiences of learning MCT principles, their understanding of the rationale and performance of techniques in sessions (*treatment receipt*), and their experiences of engaging in MCT techniques in relevant real-life settings (*treatment enactment*).

#### Treatment receipt

Treatment receipt included two aspects: patients understanding of MCT and their performance of MCT techniques during the intervention period.

Patients were asked what they thought were the main goals of MCT. Patients explained that the techniques aimed to *‘show that you have control over your own thoughts, and you can focus on what you want to focus on’* (Patient ID 3) or to practice *‘leaving thoughts alone [ … ] not interacting with them at all’* (Patient ID 4).

Patients also recognised the role of techniques in modifying their beliefs about the controllability or harmfulness of worry:

*‘[Worry postponement] definitely made me realise that I am the one in control because it’s my brain and it’s my choice whether to engage in the thought in the first place. Like, I just treat any negative thoughts now as any normal thoughts, like, because we get, like, thousands of thoughts a day and we don’t engage with every single one of them so why do we need to engage with a negative thought? We don’t need to. So, yeah, it’s definitely, like, controllable, you can definitely control it.’* (Patient ID 9).

While most young patients’ narratives showed the correct understanding of MCT and specific techniques, others misunderstood the treatment aims. For example, some patients incorrectly described the Spatial Attention Control Exercise (SpACE) to be used as a breathing technique or a tool for relaxation and distraction, even though breathing techniques, relaxation and distraction are not part of MCT. Misunderstanding the goal of techniques was also reflected by patients who described using techniques with the aim of *‘getting rid’* (Patient ID11) of their thoughts or trying to *‘push them [thoughts] down’* (Patient ID 16).

Most patients were able to describe times when they used MCT techniques within the session and how this helped them to learn ways to regulate negative thoughts or worry, for example, by leaving thoughts alone or postponing worry:

*‘I’ve been through quite a few different [therapies], but I think this one’s worked the most. [ … ] [MCT helped], like, think of ways to prevent or to postpone thoughts, and … yeah, it’s just been the best therapy I’ve had.’* (Patient ID 10)

Some patients also reported that engaging in the techniques helped them discover that worrying is controllable and/or harmless, with one patient emphasising that they found monitoring the change in their metacognitive beliefs using the belief ‘thermometers’ as particularly helpful in the sessions:

‘*I remember we did beliefs we already had [ … ] that was interesting because over the weeks, you saw your beliefs change and stuff and that was helpful to do that kind of thing.’* (Patient ID 3)

#### Treatment enactment

The second subtheme encompassed CYPs experiences relating to two components of treatment enactment: (1) their experiences of performing MCT techniques in applied settings and; (2) self-perceived benefits of receiving group-MCT.

Most of the patients who received treatment were able to provide examples of where they applied techniques such as SpACE, detached mindfulness, and worry postponement experiments outside of the sessions. For some patients, engaging in these techniques became easier and less effortful over time, with one patient describing worry postponement as becoming their *‘first instinct’* (Patient ID 13) to manage their worry. Patients also recognised and contrasted the usefulness of techniques in comparison with their old patterns of thinking:

*‘I guess, [ … ] what I used to do before is, like, if I had a negative thought then it was like, it would take up a lot of my time and I’d have to address it right then and there. But, like, just the idea of worry postponement it really helped because then I had, like, a certain time to deal with it. But when I got to that time, they were all gone anyway, so I didn’t have anything to worry about.’* (Patient ID 9)

Nevertheless, CYP did report it was challenging to remember to use techniques before getting *‘back into [their] old habits’* (Patient J) of coping, which had previously involved the use of distraction or reassurance seeking:

*‘Like, if I dwell on things, [ … ] I distract myself but … even though you’re not supposed to do that, but if it gets really bad then … and I don’t stop thinking about it, then [ … ] I will maybe message my friends’* (Patient ID 12)

Despite describing some challenges in applying MCT techniques, CYP reported that MCT improved their wellbeing, increased their confidence, and made them feel more able and willing to spend time socialising and engaging in activities that interested them. They also reported feeling happier, calmer, and more in control of their thoughts/worry, with one patient emphasising that *‘[MCT has] shown me, made me realise how powerless negative thoughts are, like, they literally, compared to, like, literally anything in the world, they are weak and can’t do anything.’* (Patient ID 10). For many, this shift in their beliefs about the power and controllability of worry (i.e. a metacognitive level change) was seen as important in reducing their anxiety and/or negative thoughts:

*‘This therapy has really helped me to just not engage in it [worry] and, like, it’s made me feel like I’m in control of my thoughts and what I do with those, so … yeah. [ … ] I was definitely more of a relaxed person and I had, like, a mindset of I don’t care anymore, if that makes sense, like, I’m the one in control so I don’t care, like, these thoughts don’t bother me anymore because I don’t have to think about them.’* (Patient ID 9)

There were, however, a few exceptions to the above where three patients (ID 5,6,16) described the intervention as unhelpful and they could not report any improvements in their thinking or emotions since the therapy ended.

### Theme 2: attitudes towards delivery

The second theme included two subthemes related to patients’ attitudes towards the format of group-MCT and the characteristics of the MCT therapists.

#### Format of therapy

Patients described that the group format of the intervention aided with their understanding and experience of MCT. Patients noted that they found it helpful to be in a group with other people who were experiencing different anxiety disorders or negative thoughts. Some reported that it helped them identify different solutions to their problems by seeing how others might solve their own issues, and others found that learning about the experiences of others with mental health problems normalised their own difficulties and made them ‘*realise that everyone feels the same way’* (Patient ID 2). CYP also described how the group format facilitated more discussion and explanations of the techniques being taught, which helped with developing their understanding of the therapeutic techniques.

CYP appreciated the small group size (average group size of six participants), with many expressing a preference for group delivery when asked if they would have preferred a one-to-one session:

*“I preferred [the group format] because it was like, not all the focus was on me and we were all doing the same things and learning the same things and you got to do it together and share each other’s ideas … I think it was good in the sense that you get a bit of, a taste for, like, other people’s past and experiences with mental health issues.”* (Patient ID 10)

Nevertheless, patients also reported negative attitudes towards the group format of therapy. For example, some patients described the group format as ‘unhelpful’, ‘intimidating’, or ‘awkward’. They reported that they found it difficult to speak out loud during therapy when there were others in the room who they did not know. Despite this, it was recognised that developing a sense of group cohesion was something that happened over time as the intervention progressed:

*‘I thought it was a lot harder to talk. [ … ] Because you’re always worried about what other people are thinking about what you’re saying as well. So, it’s harder to be more honest. [ … ] I think at the start, it was harder, yes but I think after a few weeks, it wasn’t too bad. [ … ] People just got more comfortable and then it became more of a collective.’* (Patient ID 2)

#### Therapist characteristics

Patients expressed positive attitudes towards the therapists, describing them as being friendly, helpful, and reassuring. They reported that therapists were good at explaining MCT techniques and answering any questions they had. They also described how therapists were skilled at facilitating discussions and encouraging them to participate even when they did not feel comfortable speaking out loud. Patients felt that the therapists made an effort to *‘understand what [they] were saying’* (Patient ID 10) and helped them to communicate what they were thinking or feeling when they *‘couldn’t get the words out’* (Patient ID 10) by *‘asking little questions, like one word answers, and then they put it all together and get the answer’* (Patient ID 14). In particular, it was seen that having two therapists deliver sessions was useful in helping develop patients’ understanding of MCT by having them explain the techniques in different ways:

*‘It’s like, well, they kept on switching between who, like, explained what – which I thought was quite nice because they had different ways of explaining things so, like … because sometimes they could see that we were all puzzled so they’d explain it in a different way so that was, yeah, it was quite nice.’* (Patient ID 9)

There were a few cases where patients believed a therapist failed to target their presenting problem. For example, some patients described that the questions they were asked were *‘one size fits all type [of questions]*’ and ‘*weren’t tailored to what they were going through’* (Patient ID 16). One patient highlighted that they felt that sessions were more focused on *‘how to deal with sad feelings instead of anxiety’* (Patient ID 6), and this hindered their ability to get involved in discussions. One patient also highlighted feeling uncomfortable with the style of questioning that was used, describing sessions as feeling like an “interrogation”.

### Theme 3: experiences of homework

The third theme concerned factors relating to CYP’s difficulties and successes with homework engagement and their suggestions for improving homework compliance.

Generally, patients described that they found the homework useful and that the amount given was manageable. For patients that engaged with homework, they described how it was *‘easy to do and helped’* (Patient ID 13) and how they were able to apply homework *‘without even realising it’* (Patient ID 12). Others noted that the more they engaged with homework, especially in anxiety inducing situations, the better they were able to manage their emotions: *‘I just feel like the more I did it, the more like, when I was in panicky situations, I could deal with it better.’* (Patient ID 1).

An important factor which may have influenced homework compliance was patients’ strategies used towards homework engagement. For example, some patients encouraged themselves do the homework by reminding themselves that there was a positive outcome at the end, with one patient describing, *‘if I don’t do this [the homework] then I’m not going to get the, like, the full benefits of it’* (Patient ID 9). Interestingly, this patient also highlighted their frustration that others in their group were unwilling to try homework:

*‘…a lot of people in the room, like, they didn’t do it. And my constant thought was, if you’re going into something like this [therapy] and you’re not doing what they’re telling you’re not going to get, like, the benefits from it. So, that’s what kind of frustrated me about it, it’s like you’ve got to give it a go, and not many people were.’* (Patient ID 9)

Overall patients noted benefits to engaging with the homework, but some expressed their frustration when these benefits were not experienced immediately. One patient described how they tried the homework once but soon after gave up as that single experience did not make a difference to their wellbeing, a response appearing to reflect unrealistic expectations about the immediacy of homework effects. This did, however, contradict others’ views who noted that *‘[the benefits of homework engagement is] a process, it’s not like an instant thing, it happens over time so you can’t just change overnight – which I think a lot of [patients] were thinking…’* (Patient ID 9).

Patients also reported several challenges with homework compliance. For some patients, simply the word ‘homework’ was off-putting as it often had negative connotations, *‘like, even just the word, it’s just awful.’ (*Patient ID 10) or reminded them of being at school which many associated with their anxiety and/or low mood, *‘it just kind of reminded me of school, which is where a lot of everything that I was going through stems from’* (Patient ID 16). Other patients described that they viewed homework as optional, and it was therefore not seen as a priority, unlike engaging in homework from school or extracurricular assignments which were given a higher priority. Furthermore, patients expressed that they lacked the motivation to engage with homework, describing themselves as *‘being lazy’* (Patient ID 11) or more often reported that they would simply forget to complete it. One patient gave a medical explanation for lack of homework completion; *‘I just remembered, I very much struggled [to] remember to do things because my brain does not like to do things that don’t give me dopamine’* (Patient ID 4). Another patient described finding it difficult to remember the potential benefits of engaging with homework, particularly in times where they felt more anxious, and this would hinder their motivation to practice the techniques:

*‘Most of the time I’d forget, or I’d be in a complete [anxious] state, and I just didn’t want to do it. [ … ] it would come to mind, but I’d say, I will do it, but I never actually do it. When I’m in a mood like that. I don’t see how it helps…’* (Patient ID 13)

Some narratives suggested that patients did not understand the purpose of homework, *‘I just didn’t get it. [ … ] I don’t get how, why I’m supposed to do it’* (Patient ID 15). This was also evident when some patients expressed that they did not have a suitable environment to engage in the homework; for example, when one patient was asked if they managed to practice the SpACE technique in between sessions, they responded ‘*Sometimes yes. [I was able to] when I was more just alone and [had] no distractions at all.*’ (Patient ID 13).

Patients provided several suggestions for ways in which they believed homework compliance could be improved: this included the use of a reward system (e.g., stickers or badges), reminder notifications, and further discussion with therapists that highlight the aims or benefits of doing homework.

## Discussion

In this study we explored children and adolescent experiences of the receipt and engagement with group-MCT and how patients experienced benefit. Overall, young people’s experiences of receiving group MCT were positive, with three overarching themes identified in narratives, which we labelled: fidelity, delivery and homework.

A fidelity subtheme, named ‘therapy receipt’, was comprised of two sets of experiences relating to the way therapy was understood and the experience of performing techniques during therapy sessions. CYP descriptions suggested an accurate understanding of core MCT principles consistent with the goals of therapy. Specifically, transcripts revealed that patients understood that their relationship with worry and negative thinking had changed and the treatment had helped them develop a greater sense of control over those processes. Such narratives were supported by a recognition of the role that specific techniques played in developing adaptive metacognitive beliefs about the control of repetitive negative thinking and the discovery of choice over responses. However, there were instances of misunderstanding the goal of MCT, which related to the belief that some techniques such as SpACE were forms of relaxation or a distraction device for suppressing negative thoughts.

With respect to the experience of techniques within sessions, most of the patients described using them when instructed, and there appeared to be an understanding of the important discovery experiences resulting from their usage. For example, patients specifically described how techniques helped them discover that worry is not only controllable but that it is harmless or powerless. Such perspectives are consistent with a core aim of MCT which is the acquisition and strengthening of more adaptive metacognitive knowledge. Fidelity subthemes also support some recommendations: 1) whilst the overall MCT message received appeared valid, some patients remained confused about the purpose of individual techniques. An exploration of (faulty) personal goals when using techniques should be undertaken with each patient and corrected where necessary; 2) the take-home metacognitive message derived from technique use should be repeatedly made explicit and reinforced for the group.

The second fidelity subtheme; ‘enactment’ relates to the use of techniques outside of therapy sessions in real-world contexts and to the perceived benefits of MCT. Techniques were described as becoming easier to use over time and with practise and there was recognition that they aided in the development of alternative and improved patterns of thinking. CYP reported that MCT improved their wellbeing, helping them feel “happier”, “calmer” and “more in control” of their thoughts/worry. In contrast, three patients reported no benefit from the intervention and did not describe any improvement in their thoughts and feelings. The implications arising from this set of reported experiences are as follows: 1) it may be useful to emphasise that real-world application of techniques improves with experience; 2) the message that techniques offer a means of developing a range of improved responses to thoughts should be emphasised; 3) unrealistic expectations about (immediate) effects on thoughts and feelings should be managed (see more under homework below).

Patient views also clustered around a treatment delivery theme, referring to the group therapy format and the characteristics of therapists. In particular, the group setting was described as helpful in facilitating understanding of treatment principles and reassuring in so much that patients discovered that they were not alone in their difficulties. The group helped in exposing participants to a wider range of solutions and the small group size was described as beneficial and preferred over one-to-one treatment. However, this view was not held by everyone and some patients experienced the group as intimidating, especially to begin with. CYP liked having two therapists in each group, who could take turns to explain concepts in different ways when required. Patients described that they felt “understood” but there was an instance described as therapists using a ‘one-size-fits-all model’. This was not experienced as meeting the patient’s needs, whilst in another case the intervention was seen as focused more on anxiety than low mood. The implications arising from this theme are: 1) transdiagnostic therapy for a mixture of presenting problems in a group format is not a general barrier for CYP; 2) there were advantages reported that could be used to promote the format and facilitate take-up; 3) therapists must pay attention to maintaining balance across the group in terms of personal patient relevance when using generic (transdiagnostic) principles.

The final theme centred on homework, presenting a set of experiences that were more negative than the preceding themes. Whilst some patients valued homework, there were clear negative attitudes expressed. Some patients objected to using the term, there were limited practise attempts and unrealistic expectations expressed about “immediate effects”. Narratives showed frustration and annoyance among patients in the group towards those patients who were not motivated to engage with homework. Homework compliance has been associated with increased treatment outcomes (27) and this is an area that the current experiences suggest requires further consideration in group MCT in CYP. The narratives suggest that it would be helpful to: 1) improve the level of understanding CYP have of the purpose of homework; 2) explore and modify unrealistic expectations about immediate effects on thoughts and feelings; 3) avoid connotations of “schoolwork” by changing the term ‘homework’ (e.g. use the term ‘project’ instead).

While the study highlights various benefits in delivering group-MCT to young people, and provides a patient voice to understanding therapeutic experiences, it is not without limitations. There were a greater number of females that took part in interviews in comparison to males, which makes it challenging to know if there are any sex-based differences in experiences in group-MCT. This balance is however not unsurprising as engaging young men in mental health research is a known barrier (28). Although the sample size is sufficient for qualitative analysis, the lower age of 11 years means we do not know how younger children might experience group MCT and this remains an interesting question.

In conclusion, an exploration of experiences of receiving group MCT revealed important themes consistent with the fidelity of treatment in areas of receipt and enactment in 11–17 year-olds with common mental health problems. The group format was valued (with a few exceptions), and dual therapists were seen as facilitative. CYP accounts of their experiences suggested that treatment helped them develop theoretically valid internal models of thinking and alternative strategies of mental regulation that are consistent with purported mechanisms of MCT. The results emphasise themes/areas that could be enhanced and we present a series of useful recommendations for future research and practise.

## Data Availability

The datasets presented in this article are not readily available because they are interview transcripts that participants have not consented to circulate. Requests to access the datasets should be directed to adrian.wells@manchester.ac.uk.
